# 3D Printing of Oil Paintings Based on Material Jetting and Its Reduction of Staircase Effect

**DOI:** 10.3390/polym12112536

**Published:** 2020-10-29

**Authors:** Jiangping Yuan, Chen Chen, Danyang Yao, Guangxue Chen

**Affiliations:** 1 State Key Laboratory of Pulp and Paper Engineering, South China University of Technology, Guangzhou 510640, China; yuanjiangping2009@126.com (J.Y.); chenchen_1224@126.com (C.C.); dyyao9722@163.com (D.Y.); 2 Institute for Visualization and Data Analysis, Karlsruhe Institute of Technology, 76131 Karlsruhe, Germany

**Keywords:** material jetting, 3D printing, oil painting reproduction, staircase effect, surface quality

## Abstract

Material jetting is a high-precision and fast 3D printing technique for color 3D objects reproduction, but it also suffers from color accuracy and jagged issues. The UV inks jetting processes based on the polymer jetting principle have been studied from printing materials regarding the parameters in the default layer order, which is prone to staircase effects. In this work, utilizing the Mimaki UV inks jetting system with a variable layer thickness, a new framework to print a photogrammetry-based oil painting 3D model has been proposed with the tunable coloring layer sequence to improve the jagged challenge between adjacent layers. Based on contour tracking, a height-rendering image of the oil painting model is generated, which is further segmented and pasted to the corresponding slicing layers to control the overall printing sequence of coloring layers and white layers. The final results show that photogrammetric models of oil paintings can be printed vividly by UV-curable color polymers, and that the proposed reverse-sequence printing method can significantly improve the staircase effect based on visual assessment and color difference. Finally, the case of polymer-based oil painting 3D printing provides new insights for optimizing color 3D printing processes based on other substrates and print accuracy to improve the corresponding staircase effect.

## 1. Introduction

Color is a core attribute of physical 3D objects, and its accurate reproduction becomes a tough challenge in additive manufacturing in recent years [1,2,3,4]. The color 3D printing is an advanced stage of the additive manufacturing process with vivid surface texture reproduction, which provides more authentic features of functional 3D fabrications in various industries [5]. Material jetting is one of seven additive manufacturing techniques with polymer-based materials according to the ISO/ASTM 52900 [6]. In addition, since the release of a UV inks jetting system based on the material jetting principle, its use in color 3D printing has been explored from three primary inks to six primary inks and even more spot color inks [7]. Meanwhile, the demand for high-fidelity oil paintings is increasing, but traditional manual copying methods and graphics printing techniques are difficult to reproduce the microscopic details of an oil painting quickly and accurately [8,9]. These methods easily ignore the fact that any oil painting is a 2.5D/3D object with variable thickness, thus losing the valuable signatures from the various microscopic variations in the digital acquisition of the oil painting [10]. With the launch of microscopic 3D scanning systems and the optimization of acquisition algorithms, 3D scanning data of oil paintings can be accurately acquired in a short period of time [11]. However, due to the complex color information contained in the color 3D model formed from the 3D scan data of the oil painting and the maximum print accuracy limitations of current color 3D printers, the color boundaries of the reproduced oil painting suffer from the sticky staircase effect, which destroys the high fidelity [12,13,14].

The staircase effect of 3D-printed parts is a ladder-like visual phenomenon, also called the jagging effect or terrace phenomenon, which is caused by the different boundaries of adjacent layers when sliced layers are stacked on top of each other [15]. There have been many studies on the quality evaluation and optimization of 3D-printed parts in recent years, but surface quality research is still insufficient [16]. Those surface properties are mainly the color, texture, gloss, transparency, and smoothness [17]. For example, fused deposition modeling (FDM) 3D-printed products often need to be measured for surface smoothness because its staircase effect is more pronounced [18,19,20]. An interesting study was to first fabricate the target shape with an FDM 3D printing process and then spray UV color inks on its surface for external coloring [21]. At last, they diminished the jagging effect of printed right-angle ladder models by controlling the white ink thickness and the inclination angles of the adjacent layers. Currently, the stereolithography (SLA) 3D-printed objects have higher layer accuracy and smaller surface smoothness than those printed by FDM processes, but this issue can also be found from those samples generated by contour algorithms or led by specific printing directions [22,23]. However, the buildable size of SLA 3D printers is smaller, especially with the more advanced digital lithography projection (DLP) process, which is based on polymers [24,25]. In addition, as another commonly used high-quality color 3D printing technology, the binder jetting technique consists of color binders and filled powders, even though its staircase effect has been rarely studied in color reproduction [26]. Its powder-based support materials are not suitable for the accurate composition reproduction of oil paintings, because the material reproduction of oil painting is also a challenge that affects accurate color or appearance reproduction [27,28,29]. Therefore, only the material jetting remains to make it possible to reproduce oil paintings with all color types and sizes. The material jetting techniques generally consist of continuous inkjet printing and drop-on-demand inkjet printing [30]. The PolyJet 3D printer belongs to the former, and the Mimaki UV inks jetting system belongs to the latter. The staircase effect of color 3D-printed objects is not only influenced by the colored material rendering but also by the assignment of the colored material to each layer and the color transmission method among all printed layers [31,32,33].

Currently, the effect of the colored layer features on the color reproduction of polymer-based 3D color test charts was investigated by using the PolyJet color 3D printer [34]. In that study, the authors demonstrated the qualitative correlation between the colored layer sequence and chromatic aberration, which together with the colored layer thickness affected the final color reproduction quality of the 3D-printed products. In addition, when a color 3D model is sliced with tunable thickness, the 3D printing system is defaulted to input all sliced layers with consecutive numbers in the normal order of the actual print orientation [35]. This compiled information cannot be modified by the customer during the color 3D printing process, and it is not always optimal for accurate color reproduction [36,37]. Since oil paintings are usually considered 2.5D color models and their height does not exceed 50 mm, thus, the number of layers in the overall slice is limited [38]. This provides a physical test basis for an adjustable layer thickness and variable layer order of slices or layers, which is combined with a Mimaki UV inks jetting system with variable thickness printing. Therefore, in order to achieve an accurate 3D-printed oil painting, this study proposed a comprehensive framework for its physical visualization and reduction of the corresponding staircase effect. For the physical visualization of the oil painting, we compared the staircase effect differences between the blank 3D model and the color 3D model; in terms of the oil painting’s staircase effect improvement, we analyzed the details of the oil painting reproduction of sliced layers under the orthogonal order and the colored layer customization, combining with the colorimetric evaluation. This work will enhance the color reproduction quality of polymer-based color 3D printing for new vivid applications, as well as expand accurate color reproduction understanding to develop chromogenic polymers in additive manufacturing.

## 2. Experimental

**Figure 1:**
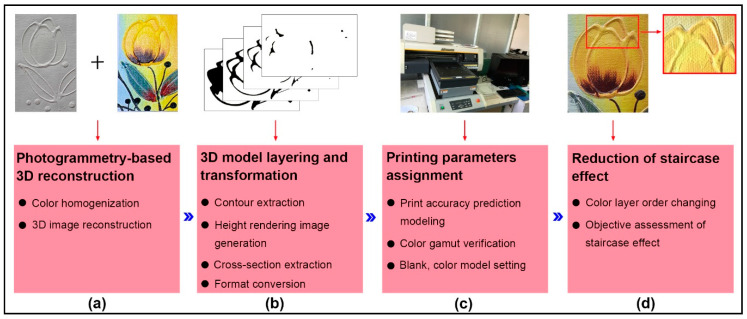
The framework of our proposed method for a 3D-printed oil painting.

The quality of a 3D-printed oil painting is easily influenced by its acquired color 3D model and process parameters. In this study, we proposed a new method to reproduce the vivid oil painting by the Mimaki UV ink jetting system and to optimize its jagged effect by adjustable colored layer sequence with a computational thickness. The framework of this method is shown in Figure 1. There are four parts, including photogrammetry-based 3D reconstruction, 3D model layering and transformation, printing parameters assignment, and optimization of the staircase effect. The Mimaki UJF-3042 UV inkjetting system (Mimaki, Tokyo, Japan) is for the output of a scanned 3D oil painting with the blank mode and color mode. LUS-120 series UV inks consist of Cyan (C), Magenta (M), Yellow (Y), White (W), and Black (K). Although support material is required for polymer-based 3D printing, the vertical placement of the oil painting model can usually be considered a positive pyramid structure. Since this structure exhibits a larger area in the lower layer than the upper layer, no additional support design is required. The oil canvas is generally made of linen, coarse fiber, more texture, and unbleached treatment. Other key tools include Ultimaker CURA slicing software (Ultimaker, Utrecht, The Netherlands), a PARAM CHY-C2 thickness gauge (Labthink, Jinan, China), an X-Rite 530 densitometer (X-Rite, Grand Rapids, MI, USA), and a Lensphoto multi-baseline photogrammetry system (Huayu Century, Wuhan, China).

### 2.1. Photogrammetry-Based 3D Reconstruction

**Figure 2:**
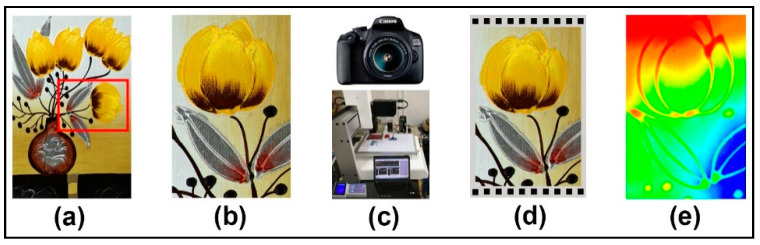
Diagram of 3D reconstruction of a specific area in an oil painting: (**a**) original oil painting; (**b**) target area; (**c**) key devices; (**d**) control grid array; (**e**) height contour rendering.

Photogrammetry, as a new contactless three-dimensional measurement with accurate geometric and color information acquisition, has been developed and industrialized by Prof. Zhang Zuxun et al. at Wuhan University with the Lensphoto multi-baseline photogrammetry system [39,40]. For the three-dimensional digitization of an oil painting, there are two main aspects: one is the accurate restoration of geometric information on the surface of the oil painting; the other is the color reproduction of the oil painting. The Lensphoto multi-baseline photogrammetry system can either obtain a digital model of the geometric surface of an oil painting or restore the color image and plane geometric position of an oil painting in the form of an orthophoto image, thus achieving a three-dimensional digital representation of the color of the oil painting. The photogrammetry-based 3D reconstruction of a yellow flower in an oil painting is illustrated in Figure 2.

A Canon 5D II SLR camera with a 50 mm solid focal length fixed lens was used for this study. Based on a calibrated oil painting reference with a control grid array, the camera’s internal orientation elements checked by the Lensphoto system were used to correct the main point coordinates and correct lens distortion. A control grid array with 40 mm intervals was used as a control plane for the oil painting recovery. Each grid size was 10 mm × 10 mm black squares with the measurable accuracy of 0.1 mm, as shown in Figure 2d. Since photogrammetry and camera CCD (charge-coupled device) color acquisition may cause color differences between adjacent acquired images, color homogenization is required prior to the 3D image reconstruction. The Wallis Transform is a local image transformation commonly used in image fusion or matching, where the mean and variance of grayscale between different images or different locations of the same image are calculated and equalized [41]. Auto-matching is performed based on the solved coordinates of the internal and external orientation elements and the feature points between calibrated images. After image matching and decoding, the point cloud model formed by the object points is further generated into a triangulated irregular network (TIN) model. This digital model can turn a captured oil painting surface into a continuous grid, greatly increasing visualization and facilitating subsequent processing such as contour tracking and rendering, as seen in Figure 2e. This height contour rendering is determined by the height range of each color feature. In addition, the original digital model format of the oil painting can be further converted to various common 3D model formats with color information, such as “.wrl”,”.dxf”, “.obj”, and “.3ds”.

### 2.2. 3D Model Layering and Transformation

Since the material-jetting type 3D printer used in this study is a specific UV ink-jet printing system, it is not possible to directly utilize the “.stl” or “.obj” formats that are commonly used for 3D printing, so manual layering is required for a digital 3D model of the oil painting. The layering process and transformation of a 3D oil painting model were here designed as four key steps: contour extraction, height rendering image generation, cross-section extraction, and format conversion.

**Figure 3:**
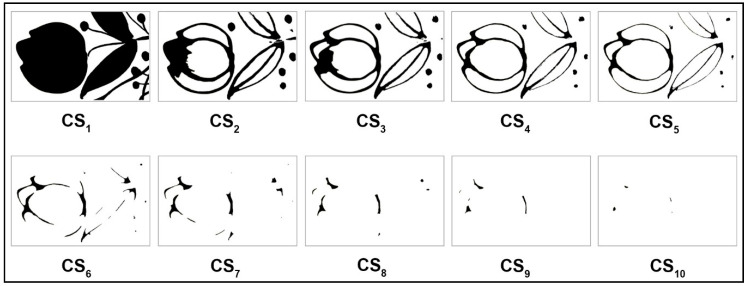
Cross-sections extraction of an oil painting model with blank mode.

Currently, the measured average printed thickness of C ink is 11.0 μm, the average printed thickness of M ink is 13.2 μm, the average printed thickness of Y ink is 7.9 μm, the average printed thickness of K ink is 9.0 μm, and the average printed thickness of W ink is 13.7 μm. The difference in thickness of the different color inks is mainly due to their specific pigment density. As the pigment density in the ink increases, the thickness of each colored layer increases. Viscosity is also a positively correlated variable, but there is a limit value for each color ink. The current viscosity of color inks is controlled in the range of 15–30 cP (25 °C) from the ink company, but no specific value is measured in this experiment. Since the minimum thickness of a single sliced layer is determined by the minimum resolution of printable accuracy and the limit of the human eye, the thickness of the sliced layer is consequently set to 100.0 μm. The maximum thickness of the selected oil painting is 1240.0 µm. The purpose of generating the contours of an oil painting model was to extract contours of different height sections for model layering with the above-mentioned thickness. The height rendering image generation was to assign relative hues and tones to each extracted height contour rendered by jetting UV inks, as shown in Table 1. Subsequently, each cross-section was segmented and extracted from this rendering consisting of ten coloring layers, which are illustrated in Figure 3, and saved as the “.eps” format file that can be recognized by the Mimaki UV inks jetting system.

### 2.3. Color 3D Printing of Digital Oil Painting Models

After each coloring layer of the oil painting model is created, it is numbered consecutively to facilitate the manual adjustment of the subsequent coloring layer print order. In this process, two printing styles were adopted: one was to print the whole shape using color inks, and the other was to print each color contour at the top layer and just print white ink in order from the bottom to the top at the other remaining layers. Setting the print distance to 2000.0 µm and the thickness of each white ink layer to 13.7 µm allowed seven consecutive layers to be printed to reach a single sliced layer of 100.0 µm, until the 13 sliced layers of this oil painting were completely printed in the normal sequence. In addition, the overall thickness of the specific printed layers was measured for all primary inks using the PARAM CHY-C2 thickness gauge. The chromatic values of 3D-printed oil paintings were measured by an X-rite 530 densitometer for the color gamut validation and color difference calculation.

### 2.4. Printing Optimization for Staircase Effect of Oil Painting Models

A new method of reverse-sequence printing of coloring layers was proposed to improve the staircase effect on the printed surface of oil paintings. The change from the normal sequence printing method was that the white ink was printed from the top layer and then the bottom layer; all other printing parameters were the same. Thus, the above three specimens were placed under a lamp cabinet to subjectively evaluate the reproduction effect of the yellow flower oil painting, and the areas with significant edge changes were selected for objective evaluation of the staircase effect utilizing the color difference metric. The CIEDE*_ab_ formula is to acquire the chromatic aberration of two colors on 3D-printed oil painting [42], which is shown in Equation (1).

(1) $$\Delta E_{\mathrm{ab}}^{*}=\sqrt{{\left(L_{t}^{*}-L_{o}^{*}\right)}^{2}+{\left(a_{t}^{*}-a_{o}^{*}\right)}^{2}+{\left(b_{t}^{*}-b_{o}^{*}\right)}^{2}}$$

The $L_{o}^{*}$, $a_{o}^{*}$, and $b_{o}^{*}$ are the original chromatic values, and the $L_{t}^{*}$, $a_{t}^{*}$, and $b_{t}^{*}$ are the target chromatic values. They are both measured data from the printed samples or acquired sample images.

## 3. Results and Analysis

In this section, there are three aspects: the first is that the correlation between the printed ink thickness and successive layers are used to determine to determine the slice thickness of the oil painting model and the order in which the coloring layers can be assigned; the second is the physical visualization of three photogrammetry-based oil painting models; the third is the effect of the proposed method on the staircase effect in the test area of this oil painting.

### 3.1. The Correlation between the Printed Ink Thickness and Successive Layers

**Figure 4:**
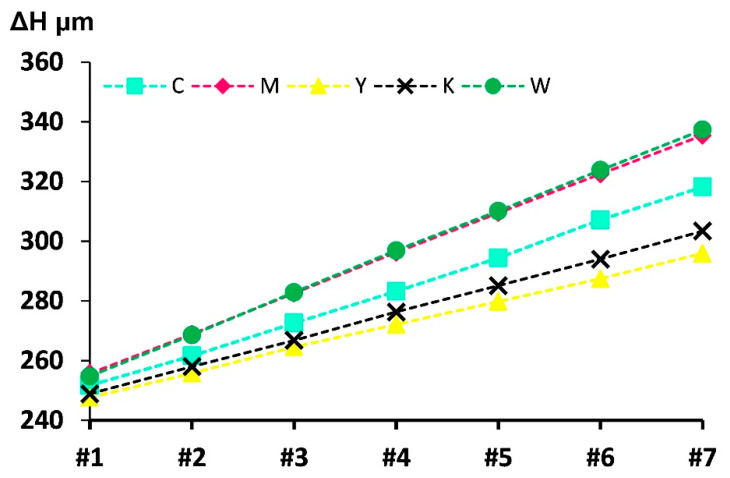
A diagram to quantify the printed ink thickness and successive layers.

Figure 4 shows seven samples that were 3D-printed using respectively five primary inks including C ink, M ink, Y ink, K ink, and W ink. Each sample corresponds to the slice layer number of the oil painting model described in Section 2.2. For sample #1, it means the first sliced layer with nine printed ink sheets. By analogy, sample #7 implies seven consecutive slice layers containing 63 printed ink sheets. From the slope of each linear fitting line, it is clear that the printed layer thickness of each primary ink of the Mimaki UV inks jetting system can be consistent at the microscale for different height models. This feature demonstrates that the current primary inks fulfill the criteria for the accurate printing of each slice layer of the photogrammetry-based oil painting model, and that the tight connection between the color ink layers can be controlled. Meanwhile, since each sliced layer contains nine ink layers, the entire linear trend in seven models with different sliced layers confirms that the sequential switching of the coloring layers in each slicing layer does not affect the print accuracy of the entire sample. Moreover, this provides a theoretical basis for the subsequent optimization of the staircase effect of 3D-printed oil paintings.

### 3.2. Physical Visualization of Three Photogrammetry-Based Oil Painting Models

**Figure 5:**
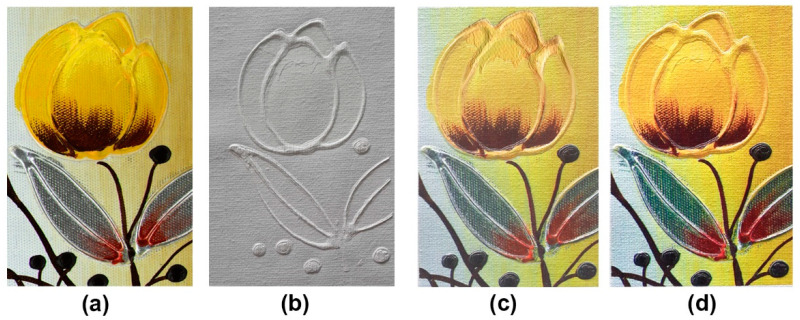
The visualization difference between digital original source and 3D-printed replicas: (**a**) photogrammetry-based 3D model; (**b**) blank 3D model; (**c**) regular color 3D model; and (**d**) revised color 3D model.

Figure 5 shows the final physical visualization results of three photogrammetry-based oil painting models, such as the blank 3D model with white ink printing, the regular color 3D model with normal sequential printing, and the optimized color 3D model with reverse order printing. From the blank 3D model in Figure 5b, the staircase effect is evident where the curvature varies greatly in its entire surface height trend, and it is also implicit where the curvature varies unevenly. Since the monolayer thickness of both the color ink layer and the white ink layer is different, these areas become more visible in the normal sequence 3D-printed color model, as shown in Figure 5c. Since the current printing system is based on planar forming, rather than liner printing, each small sliced layer with certain ink layers can be precisely formed on a larger sliced layer with specific ink layers, thus showing sharp boundaries between adjacent sliced layers. Obviously, in the 3D-printed oil painting model shown in Figure 5d with our proposed reverse-sequence method, the staircase effect of the corresponding positions is not easily detectable by the human eye. This can be explained by the actual phase change characteristics of the jetting droplets array; for instance, some droplets in upper ink layer with planar droplets array are slightly flowing due to the lack of support prior to sufficient solidification [43,44], thus easing the adjacent boundary between the upper large ink layer and the lower smaller slice layer.

### 3.3. Effects of Reverse Order Printing Method on the Jagged Issue of 3D-Printed Oil Painting

**Figure 6:**
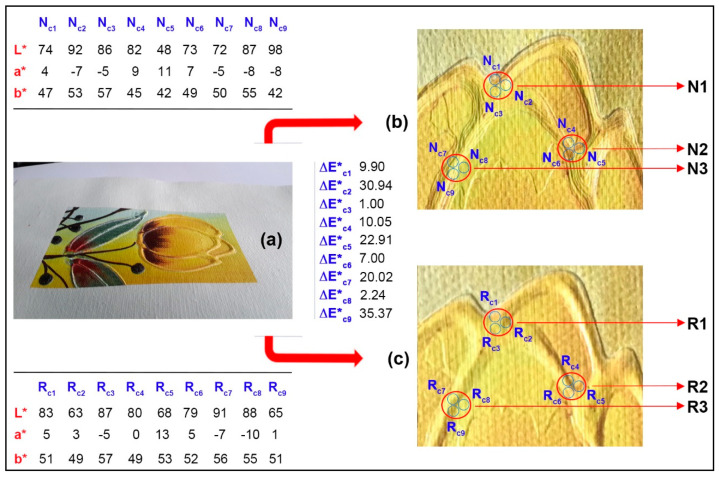
Local block comparison of two oil painting replicas: (**a**) complete sample; (**b**) a local block of normal-sequence sample; (**c**) a local block of reverse-sequence sample.

Subjectively and visually, the staircase effect of the improved oil painting model was indeed reduced, but the quality of color reproduction in the corresponding regions needs further objective evaluation. In Figure 6, three featured areas marked with big red circles are selected for comparison in the same locations on each local block of the two oil painting replicas. In addition, three additional small blue circles were selected within each large red circle for the colorimetric measurements. In addition, the corresponding measured CIEL^*^a^*^b^*^ values for nine sampled color points are both shown in the upper left and lower left corners of Figure 6, respectively. It is noticeable that the chromatic values of the sampled points in the same red circle area are also different, which also matches the color differences on the different staircase layers.

Figure 6 also showed chromatic aberrations between the upper and lower color sample points; the smallest chromatic aberration can be found within sampling blue area 3, and the largest chromatic aberration can be found within sampling blue area 9, but the overall average chromatic aberration is 15.49. For the whole area 1 and area 2, which are both marked with a big red circle, the corresponding average chromatic aberrations are approximate (the former is 13.94, the latter is 13.32), while the color difference in the red area 3 changed sharply (the value is 19.21). There are two areas (blue area 2 and blue area 9) where the chromatic aberration exceeds 30, but these are not apparent to visual observation, which may be due to the complex light mixing at the boundary of the staircase effect area. In summary, chromatic aberration control is also extremely important in areas where the staircase effect is pronounced, and it requires further comprehensive research for more accurate reproduction based on reverse-sequence printing model.

## 4. Discussion and Conclusions

For oil painting replication, our team has implemented key techniques ranging from the digital 3D model acquisition of oil paintings to their color 3D printing, and it has also proposed a specific series of methods for accurate color reproduction [8,10,12,36]. An important contribution of this paper is to provide a comprehensive framework to the issue of staircase effects arising from the 3D reproduction of oil paintings. The chromaticity evaluation in the region where the staircase effect is evident is also the first attempt, which provides an objective metric for the accurate reproduction assessment of oil paintings printed by the polymer-based color 3D printers. Moreover, this study also extends the application of coloring layer sequence to the realistic reproduction of surface features in color 3D-printed models, although the mechanism of this effect is not yet clear in the field of color 3D printing.

For the polymer research or polymer-based additive manufacturing community, this article also has some limitations that need to be explored further. Firstly, this high-precision color 3D printing system has taken advantage of advanced array jetting techniques in graphics printing, and it is capable of multi-material (ink) injection and droplet accuracy under 16 μm while printing models within 5 cm in height. If the final printed 3D model exceeds this height, it will deform from our predictive experiments. Other polymer-based material jetting type 3D printers have not been able to achieve this scale from commercial machines worldwide [45]. In Section 2.3, the printing parameters given in this article have been tested experimentally to ensure the accuracy of the shape printing of the oil painting model. For this reason, there is no comparative analysis of the material properties of UV-curable oil paintings, which are of interest to developers of polymer-based inks. For example, the references [27,29] discussed the materials component identification of oil paintings by analyzing UV-vis spectroscopy, reflection FT-IR spectroscopy, mass spectrometry, and chromatography, but none of them are sampled at the boundary of the coloring layers. In addition, modifying droplet parameters for the color inks may be a regular way for polymer research to increase print resolution and remove the staircase effect, such as pigment dispersion, curing time, surface tension, and so on.

Secondly, when analyzing the chromaticity of the oil painting replicas, we can see that there are large variations of color differences in the same local boundary areas. These variations do not show a unified linear trend in quantification, so we chose the average value to assess. In addition, the objective evaluation of the color reproduction of 3D-printed parts can also be performed without contact using the mean structural similarity (MSSIM) metric of captured images of the sample surface [34]. Other image quality metrics are also explored for FDM-type 3D-printed models, such as Hough transform, histogram equalization, and entropy of depth maps [46,47]. The combined assessment of these objective metrics has also been discussed in the regular print sample and may provide a more comprehensive view of the 3D-printed oil painting reproduction [48].

Thirdly, the existing coloring layer sequence adjustment is manual and therefore not yet efficient for extending the proposed framework to other polymer-based 3D printing processes. This deficiency can be addressed technically by embedding it into the slicing process, but the determination of the thickness and coloration of the slicing layer still requires physical testing and subjective evaluation by the observer based on our proposed framework. For example, whether the current reverse-sequence printing method is also effective for polymer-based color 3D printing systems with slice layer thicknesses greater than 100 µm, and the choice of global or local reverse-sequence deserve further testing to verify in the fields of oil paintings or topographic maps. In addition, the development of spot-color inks is concerned with the curing accuracy of the coloring polymers, so that the spot-color inks are as close as possible to the thickness of the primary inks in a single printed layer, thus simplifying the selection of the sequence of coloring layers for color model slicing.

## Figures and Tables

**Table 1:** Coloring feature of each layer contour for an oil painting model.

Height (μm)	Color Feature	R	G	B
0–100	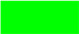	0	255	0
100–200	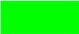	50	255	0
200–300	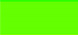	100	255	0
300–400	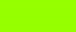	150	255	0
400–500	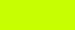	200	255	0
500–600	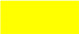	255	255	0
600–700	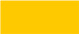	255	200	0
700–800	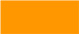	255	150	0
800–900	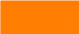	255	125	0
900–1000	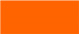	255	100	0
1000–1100	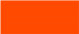	255	75	0
1100–1200	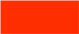	255	50	0
1200–1300	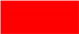	255	0	0

